# Inequitably harmed: a scoping review protocol on patient safety and diverse population groups

**DOI:** 10.1136/bmjopen-2025-108797

**Published:** 2025-12-24

**Authors:** Josephine Ocloo, Shone Surendran, Blaine Robin, Leah Noel, Jade Gibson, Shoba Dawson

**Affiliations:** 1 Centre for Mental Health Policy and Evaluation (CMHPE), Department of Health Service and Population Research, Institute for Psychiatry, Psychology and Neuroscience, King’s College London, London, England, UK; 2 National Institute for Health Research (NIHR) Applied Research Collaboration (ARC) South London, Institute of Psychiatry, Psychology and Neuroscience, King’s College London, London, England, UK; 3 Department of Health Service and Population Research, Institute for Psychiatry, Psychology and Neuroscience, King’s College London, London, UK; 4 Sheffield Centre for Health and Related Research, School of Medicine and Population Health, The University of Sheffield, Sheffield, England, UK

**Keywords:** Patients, Safety, Health Equity, Health Services, Patient Participation

## Abstract

**Abstract:**

**Introduction:**

Patient safety is a central pillar of healthcare quality. However, with repeated examples of failure emerging across healthcare, there is an ongoing need to better understand how the safety of care can be improved for patients. Evidence suggests that some population groups are more likely to inequitably experience healthcare harm. This review will look at what evidence exists on understanding patient safety harm and its causes and impact on different population groups and particularly those from marginalised backgrounds. It will also focus on what actions can be taken to address patient safety disparities and service improvements, including with patient and public involvement.

**Methods and analysis:**

A scoping review of empirical and grey literature will be conducted following the Joanna Briggs Institute guidance. Medical databases such as Medline, EMBASE, PsycINFO will be searched for peer-reviewed articles and grey literature sources such as BASE, institutional and government repositories will be searched for reports, independent reviews, confidential enquiries, etc. These will be searched from 2001 to present for publications in English. Title and abstract and full text screening will be undertaken by one or more people acting as first reviewers and validated by a second reviewer. A data extraction form will be used to extract data including equity considerations following the PRO EDI framework. Data will be grouped thematically and analysed using a narrative approach.

**Ethics and dissemination:**

Ethics approval is not required for this work as the information used is publicly available. The findings of the review will be disseminated through stakeholder meetings, a peer-reviewed publication and conference presentations.

**Protocol registration number:**

osf.io/4mfus.

**STRENGTHS AND LIMITATIONS OF THIS STUDY**

- The review will cover both peer -reviewed and grey literature on patient safety.
- The review will be coproduced with experts by occupation and experience.
- The review will report on equity considerations based on the PRO EDI framework.
- A limitation is that we will only include studies published in English.

## Introduction

 Patient safety is a central pillar of healthcare quality^1^,^3^and defined as the ‘avoidance, prevention and amelioration of adverse outcomes or injuries stemming from the process of healthcare’.^4^ Nearly 1 in 10 patients is harmed in healthcare, translating to over three million deaths globally each year and costing health systems billions of dollars each year. More than half of this harm is deemed preventable and seen as a shocking call for major systemic change.^5^ In the UK, with repeated examples of failure emerging across healthcare, Bristol Royal Infirmary Inquiry^6^; The Mid Staffordshire National Health Service (NHS) Foundation Trust Public Inquiry^7^; Mazars Report^8^; Independent Medicines and Medical Devices Safety Review,^9^ there is an ongoing need to better understand how we can improve the safety of care to patients.

In healthcare, there is a robust body of evidence to show that socioeconomic disadvantage, gender and class inequities and racism are among the ‘fundamental causes’^10^ of health and disease.^11 12^ There is relatively little focus and discussion, however, in patient safety on inequities and healthcare harm impacting different population groups. Yet safety and equity can be seen as fundamental dimensions of healthcare quality. Stratified and disaggregated data are needed to identify inequities and health equity-focused prevention strategies and policy reforms to drive improvements.^13 14^ More research in this area can help to develop interventions to improve safety and equity together, and to measure the efficacy of such interventions.^15^

Increasing evidence suggests that patient safety is a serious concern for many population groups.^16^ Global reports like those from the WHO highlight higher risks of patient harm for older adults, children and ethnic minority groups.^5^ Older patients are at increased risk of patient safety harm, which has been identified as a serious concern for older patients with long-term conditions.^17^ In primary care, older patients with multimorbidity (two or more long-term conditions) are especially likely to experience patient safety incidents.^18^ Disparities have been found in the quality and safety of postdiagnostic primary care for people with dementia based on socioeconomic status (SES), particularly for a range of prescribing indicators.^19^ Unsafe care is also a leading contributor to child mortality and long-term disability, especially in the early stages of life. Reviews of paediatric care reveal harmful incidents are widespread with the most common causes of harm, medication errors, diagnostic errors, health care-associated infection, surgical complications and incidents related to medical devices.^20^

People from ethnic minority groups are more likely to report poorer health and poor experiences of health services (eg, maternity mortality, mental health) than their white counterparts.^21 22^ Ethnic minorities are vulnerable to a higher rate of patient safety events in hospital and community settings compared with the mainstream population.^23 24^ An international systematic review on the incidence of medication error (with the majority of the studies from the USA) found that ethnic minority groups are more susceptible to prescription errors, undertreatment, administration errors and suboptimal medication monitoring and follow-up by healthcare providers.^25^ Longstanding inequities in maternal mortality rates remain in the UK, with nearly a threefold difference in rates among women from black ethnic backgrounds and an almost twofold difference among women from Asian ethnic backgrounds compared with white women.^26^ The USA continues to have the highest rate of maternal deaths of any high-income nation, despite a decline since the COVID-19 pandemic. Within the USA, the rate is by far the highest for black women. Most of these deaths—over 80%—are likely preventable.^27^ More intersectional experiences in this clinical area in the USA have found that across the perinatal period, respondents with moderate or severe disability experienced worse outcomes than those without disability.^28^ With respect to mental illness, this area is closely associated with many forms of inequities. Health inequities are avoidable and unfair differences in health status and determinants between groups of people due to demographic, socioeconomic, geographical and other factors. These differences can be in relation to prevalence, access to, experience and quality of care and support, as well as opportunities and outcomes. Health inequalities can mean reduced quality of life, poorer health outcomes and early death for many people.^29^

In the USA, patient safety events have been found to be positively associated with physical harm and 30-day mortality in non-psychiatric hospitalisations for persons with serious mental illness.^30^ Diagnostic inequities in autism spectrum disorder and other mental disorders also persist across US patient populations.^31^ In Sweden, psychiatric disease, including all psychiatric diagnoses, regardless of severity, has been found to nearly double the risk of being a reported case of preventable harm.^32^

Patient safety concerns about discrimination and avoidable deaths for those with learning disabilities are longstanding. International data shows people with learning disabilities experience significantly higher rates of avoidable death compared with the general population. In the UK, the NHS England Learning Disability Mortality Review Programme report for 2023 shows that 39% of deaths for people with learning disabilities were deemed avoidable (the rate is almost double that of avoidable deaths in the general population (21%). 37% of cases reported some form of delay in care or treatment, while 28% reported instances where diagnosis and treatment guidelines were not met. People with learning disabilities from minority ethnic communities die significantly younger than their white British counterparts.^33^ Wider international evidence confirms that people with learning disabilities appear to experience poorer patient safety outcomes in hospital.^34^ In New South Wales, in a study that investigated mortality and its causes in adults over the age of 20 years with intellectual disability (ID), adults with ID experienced premature mortality and over-representation of potentially avoidable deaths. After recoding deaths previously attributed to the aetiology of the disability, 38% of deaths in the ID cohort and 17% in the comparison cohort were found to be potentially avoidable.^35^

Reporting and learning from safety incidents have become a key tool in patient safety prevention work. This has led to the development of various systems for reporting serious incidents in the NHS in the UK like the Strategic Executive Information System (STEIS) and reporting patient safety incidents into the National Reporting and Learning System (NRLS). The Learn from Patient Safety Events (LFPSE) service was recently introduced by NHS England (the body that leads the NHS in England, with responsibility for planning, commissioning and delivering healthcare services across the country), as a modern replacement for both STEIS and NRLS in 2021.^36^ It allows healthcare organisations to report a broader range of incidents, offering improved categorisation to better identify trends, manage risks and enhance patient safety as part of NHS England’s ongoing safety improvement initiatives.

The recently published patient safety healthcare inequalities reduction framework^37^ recognises that ‘inequalities cause or increase the risk of harm to patients in healthcare’ and are patient safety healthcare inequalities. Viewing healthcare inequities through the lens of patient safety is seen as an important line of action for which healthcare professionals and systems have a clear responsibility.^37^ While LFPSE collects some demographic data, a key national aspiration within the healthcare inequalities reduction framework is to ‘develop the LFPSE service to record the protected characteristics of those involved in patient safety events to identify when patient harm is more common in specific groups of patients, and whether there is case selection bias in patient safety incident’ investigations.^37^

This thinking illustrates that in safety science, there is a need for a broader knowledge framework for the evaluation of medical harm and risk that is wider than a biomedical framework and based on a more patient-centred ethos.^38 39^ Central to this should be an understanding of experiences arising from systemic biases of race, ethnicity, disability, age, class, gender, sexual orientation, etc. There have been repeated calls to involve patients and the public to better improve performance and quality.^40 41^ Many groups are under-represented in healthcare improvement and safety, including those harmed by healthcare processes.^42 43^ In the patient and public involvement (PPI) literature, there is also very little information in patient safety which looks at the experiences of diverse groups in relation to equity, diversity and inclusion.

This deficit in the literature sits in stark contrast with a wider body of knowledge in the social science literature that focuses on equity, discrimination and the broader experiences of underserved groups and communities. This underscores the importance of incorporating this knowledge into patient safety, which may also help to assign accountability for learning from these inequities and for addressing them.^44^

The proposed scoping review will characterise, identify and map the available international empirical evidence and grey literature on how different groups are at risk or affected by patient safety harm and how all population groups, and particularly those from marginalised backgrounds, are impacted.

### Key research questions will include

1. What evidence exists on understanding patient safety harm (mental and physical) and its causes and impact on different population groups and particularly those from marginalised backgrounds?
2. Which groups are more likely to be harmed or to be at risk of poor outcomes due to patient safety harm and why are these groups more at risk?
3. What actions can be taken to address patient safety disparities and service improvements with different groups including PPI?

### Methods and analysis

This review will be conducted in accordance with the Joanna Briggs Institute methodology for scoping reviews^45^ and reported by following the Preferred Reporting Items for Systematic reviews and Meta-Analysis extension for Scoping Reviews.^46^

#### Inclusion criteria

Eligibility criteria are informed by the Population, Concept, Context framework as described in (table 1, along with definition of ‘protected characteristics’).

**Table 1:** : Eligibility criteria.

	Inclusion criteria	Exclusion criteria
Population	General populations or marginalised or underserved groups including ethnic minority groups, women (maternal care), older adults, disabled people, LGBTQIA+* populations. Particular attention is given to intersectionality and the compounded effects of multiple forms of disadvantage.	Studies/reports not discussing population-level outcomes or those that generalise findings without disaggregation.
Concept	Exploring patient safety events, preventable/avoidable harm/lessons learnt, systemic healthcare failure or safety improvement efforts including negligence, malpractice, safety culture and institutional responses to harm in diverse populations from a global perspective. Patient involvement in patient safety literature will be included. Address patient safety as it relates to marginalised populations and/or explore disparities in safety outcomes associated with one or more of the nine protected characteristics (as defined by the UK Equality Act 2010), including but not limited to: race/ethnicity, age, sex, pregnancy/maternity, disability, sexual orientation, gender identity, religion or belief and marital/civil partnership status.	General healthcare research or policy documents not linked to safety, risk or institutional learning. Studies/reports with no reference to population diversity or where equity dimensions are not relevant. Patient involvement in clinical decision making without patient safety considerations will be excluded.
Context	Healthcare or social care contexts, including but not limited to: primary care, secondary care, tertiary care, mental health services, community or voluntary sectors or institutional/organisational governance structures related to patient safety.	Non-healthcare context will be excluded.
Study designs	Quantitative, qualitative or mixed methods designs. Theoretical and conceptual frameworks may be included only if explicitly linked to empirical findings or if they form part of a systematic review.	Randomised controlled trials will be excluded.
Time frame	Studies/reports published from January 2001 onwards will be considered. Pre-2001 literature may be included selectively where studies/reports are of historical significance, have shaped contemporary debates or are frequently cited in more recent work.	Pre-2001 reports without citation or relevance to current patient safety discourse.
Language	Only studies/reports available in English will be included due to resource limitations.	Non-English studies will be excluded.
Sources of evidence	Peer-reviewed academic articles, public inquiry reports, independent reviews, landmark legal cases with national/international patient safety policy impact (eg, Montgomery vs Lanarkshire), confidential inquiries, whistleblowing summaries.	Unpublished internal audits, editorials, commercial industry reports, dissertations, blog posts or news articles, routine internal reviews, local operational reports and general court case judgements. Systematic reviews will be excluded.

* LGBTQIA+ is an umbrella term that refers to individuals who identify as lesbian, gay, bisexual, transgender, queer or questioning, intersex, asexual and other minoritised sexual and gender identities. The ‘+’ acknowledges the spectrum of identities that may not be explicitly listed but are part of the broader queer community.

### Search strategy

A comprehensive search strategy building on previous review was developed using blocks of terms related to patient safety and diverse population groups.^44^ It was developed and tested for sensitivity and specificity on MEDLINE using a combination of free-text and MeSH terms. It was then replicated on Embase and PsycINFO.

Additionally, grey literature will include national and international key patient safety reports, independently commissioned government inquiry, review and investigation reports. The following databases will be searched: BASE, and institutional repositories—King’s Fund, Nuffield Trust and Health Foundation. We will also search governmental repositories—UK government and parliamentary websites, national, international and NHS archives (eg, Department of Health and Social Care, parliamentary select committees, NHS England, the National Guardian’s Office, Health Services Safety Investigations Body and Ombudsman reports) and related organisational and patient safety bodies.

See online supplemental file 1 for an example search strategy. The reference lists of the included studies and reports and relevant systematic reviews will be searched for additional studies for inclusion.

### Screening

After deduplication of search results in EndNote, all citations will be exported to Rayyan for screening. Titles and abstracts will be independently screened 50% each against the inclusion criteria by two first reviewers (SS) (JG) and an overall 50% will be cross-checked and validated by a second reviewer (GS). Subsequently, full texts will be independently screened by the first reviewer (SS) and checked for validation by a second reviewer (JG). Any disagreements will be resolved through discussion or input from a third reviewer (JO) (SD).

### Data extraction

Data will be extracted into a table developed by the review team on Microsoft Word. The extracted data will include: author, year, country, publication type, population, evidence/type of patient safety harm, impact on population, patient and family perspectives of harm, evidence of explanatory factors relating to inequities and risk of harm, evidence of patient involvement in patient safety and evidence of PPI in safety interventions. From the grey literature, we will also extract evidence of whistleblowing or staff concerns, availability of patient or staff testimony, key recommendations or impact of patient safety and health equity.

To extract data on participant characteristics, we will apply the PRO EDI framework^47^ to capture equity-related considerations in reviews and identify any potential gaps. This will include age, sex, gender, sexual identity, race, ethnicity and ancestry, SES, level of education and disability. Quality appraisal will not be undertaken in line with the scoping review guidance.

Prior to commencing data extraction, the form will be piloted on a small random sample of 10 studies/reports and then modified and refined as required. Data will be extracted by the first reviewer (SS) and checked for validation by a second reviewer (JG). Any disagreements will be resolved through discussion or involvement of a third reviewer (JO).

### Data synthesis

Extracted data will be thematically grouped for further analysis using a narrative approach. Findings will be presented in tables and narratively. This will include mapping types of patient safety concerns, equity-related considerations, where evidence related to inequities and patient safety exists, and where the evidence and knowledge gaps are. We will also classify reports and studies by the nine protected characteristics under the Equality Act 2010^48^ and their relevance to PRO EDI principles. Two reviewers (SS) (JG) will synthesise this data and it will be cross-checked by a second reviewer (GS). Any disagreements will be resolved through discussion with a third reviewer (JO).

### Patient and public involvement

This review will follow a Participatory Action Research approach.^49^,^51^ This participatory approach is intended to embed experiential knowledge throughout the review and strengthen the relevance and impact of the findings. The review will be coproduced by a diverse team of academic researchers and peer researchers (experts by experience). The peer researchers will have lived experience of patient safety issues and patient harm, with representation from individuals from minority ethnic backgrounds and with lived experience and expertise in disability issues . Their involvement will span different stages of the review process where they wish to be involved, including the study design and development of the protocol, literature searching, screening, data extraction, especially with the grey literature, qualitative synthesis, analysis, write-up and dissemination of findings.

The review is part of a wider study looking at patient safety and epistemic exclusion. The review, as part of this wider study, will benefit from feedback from an advisory group connected to the wider research the scoping review is part of. A smaller working operational group of university and peer researchers will take the study forward in terms of implementation. The advisory group, made up of 20 members, brings together people with lived experience, academic researchers, public contributors and members of four regional Integrated Care Boards (ICBs). The advisory group meets three times a year and can therefore provide feedback at different stages of the review, particularly, for example, feedback in the early stages on the search strategy, research questions, screening and data extraction forms and helping to disseminate the published materials.

To support the involvement of peer researchers in this scoping review, training will be provided to them on systematic and scoping reviews and evidence synthesis. This training will be co-designed and delivered online by university researchers experienced in public involvement, offering a clear and accessible approach. Practical and interactive sessions will include building skills in screening, data extraction and qualitative synthesis. Peer researchers will be paid £25 per hour for their time and expertise. The GRIPP2 short form checklist (Guidance for Reporting Involvement of Patients and the Public, Version 2) ^52^ will be used to plan and report a clear account of the aims, methods and outcomes of PPI involvement in undertaking our scoping review.

### Ethics and dissemination

As this is a scoping review, all data are available publicly and therefore ethical approval is not required.

The final scoping review paper will be disseminated through publication in a peer-reviewed journal and will be presented to various stakeholders. This will include ICBs who are NHS organisations responsible for planning and delivering local health services within a specific geographical area and National Institute for Health and Care Research Applied Research Collaborations (who support local partnerships for applied health and care research) connected to the project and their considerable community and voluntary networks. The principal investigator (PI) for the study has various patient safety network contacts which will allow the protocol to be disseminated widely: via NHS England (the organisation that oversees the provision of healthcare services in England), harmed patients and their families and organisations supporting patients in this area like the Harmed Patients Alliance and via the WHO Global Patient Safety Network which the PI is a member of.

## Supplementary material

10.1136/bmjopen-2025-108797 online supplemental file 1
