# Supplementary material for: Inequitably harmed: a scoping review protocol on patient safety and diverse population groups
Source: BMJ Open. 2025 Dec 24;15(12):e108797. doi: 10.1136/bmjopen-2025-108797 (PMC12742063; doi:10.1136/bmjopen-2025-108797)
Supplement: online supplemental file 1 [file bmjopen-15-12-s001.pdf]

Supplementary File 1: Example Search Strategy

Database(s): Ovid MEDLINE(R) 1946 to present
Search Strategy:

#	Searches	Results
1	exp Malpractice/	32810
2	*"Patient Safety"/	13707
3	patient safety.tw,kw.	48158
4	safety culture.tw,kw.	3542
5	(safe$ adj2 (practice$ or manage$)).tw.	20509
6	(Diagnostic Errors or Patient Safety or Iatrogenic Disease or Hospital Mortality).kw.	13046
7	malpractice$.tw.	11194
8	(patient adj2 harm$).tw.	3595
9	human error$.tw.	3649
10	((service$ or system$ or communication$ or organisation$ or organization$) adj1 (weak$ or fail$)).tw.	7277
11	(latent adj1 (threat$ or cause$ or fail$)).tw.	352
12	((medica$ or diagnostic or therapeautic or administration or dispensing or prescri$) adj1 (error$ or mistake$ or fault$)).tw.	19741
13	(near miss* or close call* or Nosocomial or (Patient* adj2 safety) or adverse outcomes or adverse events or incident reports or Root Cause Analysis or RCA or sentinel event*).tw. /freq=3	42734
14	(never event* or adverse event*).tw. /freq=2	78474
15	*Medication Errors/	10047
16	(serious incident* or untoward incident*).tw,kw.	380
17	adverse event*.tw.	263063
18	*"Medical Errors"/	12457
19	negligence.tw.	4597
20	or/1-19	417023
21	Social Marginalization/ or Social Isolation/	18262
22	(marginalization or social isolation).tw. /freq=2	5098
23	Vulnerable Populations/	13873
24	(hard to reach adj1 (people or patient* or group* or population* or sample* or participant*)).tw.	1342
25	(asylum seeker* or migrant* or immigrant* or "culturally and linguistically diverse" or CALD or "Non English Speaking" or NESB or (Ethnic* adj2 patient*) or Indigenous population* or Language barrier*).tw. /freq=2	35469

26	((vulnerabl* or exclude* or segregat* or discriminat* or disadvantag* or minority or disabled or poor or low-income or isolated or frail or polypharmacy or abused or maltreat or ill) adj1 (people or patient* or group* or population* or sample* or participant* or child* or older adult* or aged or elderly)).tw. /freq=2	43452
27	Ill-Housed Persons/ or Homeless Youth/	12127
28	(homeless* or substance user* or sex worker* or refugees or asylym-seeker*).tw. /freq=2	19399
29	*Alcoholism/ or *Drug Users/	65140
30	Sex Workers/	3418
31	Refugees/	14834
32	"Transients and Migrants"/	15695
33	indigenous people.tw.	3195
34	(ethnic minority groups or racial-ethnic disparities).kw.	502
35	Ethnicity/ or "Minority Groups"/	91321
36	"Ethnic and Racial Minorities"/sn [Statistics & Numerical Data]	127
37	Racial Groups/eh [Ethnology]	826
38	Racial Groups/sn [Statistics & Numerical Data]	5081
39	Rare Diseases/ or rare disease*.tw.	46611
40	care-leaver*.tw.	71
41	looked-after child*.tw.	143
42	*"Learning Disabilities"/	10783
43	(person with disability or person with disabilities or people with disability or people with disabilities or disabled person or disabled people or intellectual disability).tw.	30399
44	cognitive impairment.tw. /freq=2	39834
45	((economic* or social* or financial*) adj1 disadvantage*).tw.	7133
46	(orphan adj1 (child or condition* or disease*)).tw.	1514
47	Child, Orphaned/	812
48	*Persons with Visual Disabilities/ or *Persons with Hearing Disabilities/ or *Deaf-Blind Disorders/	5346
49	*Deaf-Blind Disorders/	169
50	*Poverty/	19112
51	*Language/	29985
52	Social Discrimination/ or Disability Discrimination/	1857
53	(Discrimination or structural racism or systemic racism or poverty or deafblind).tw. /freq=3	27949
54	Systemic Racism/eh, sn [Ethnology, Statistics &Numerical Data]	59
55	*Low Socioeconomic Status/ or *Socioeconomic Factors/	15141

56	*"Health Literacy"/ or exp Health Inequities/ or Health Status Disparities/ or Health Equity/ or Healthcare Disparities/eh [Ethnology]	60745
57	*"Mental Health Services"/ or *Inpatients/ or (mental health services or mental illness or mental disorders).kw.	58447
58	or/21-57	619857
59	20 and 58	6597
60	limit 59 to english language	6342
61	limit 60 to (address or autobiography or bibliography or biography or case reports or collected work or comment or congress or consensus development conference or consensus development conference, nih or dataset or dictionary or directory or editorial or festschrift or guideline or interactive tutorial or interview or lecture or legal case or legislation or letter or news or newspaper article or observational study, veterinary or observational study or overall or patient education handout or periodical index or personal narrative or portrait or practice guideline or preprint or randomized controlled trial, veterinary or "review" or video-audio media or webcast)	1297
62	60 not 61	5045
63	limit 62 to dt=20010101-20250710	4639
