# Supplementary material for: Inequitably harmed: a scoping review protocol on patient safety and diverse population groups
Source: BMJ Open. 2025 Dec 24;15(12):e108797. doi: 10.1136/bmjopen-2025-108797 (PMC12742063; doi:10.1136/bmjopen-2025-108797)
Supplement: Supplementary file 2 [file bmjopen-2025-108797.reviewer_comments.pdf]

PEER REVIEW HISTORY

BMJ Open publishes all reviews undertaken for accepted manuscripts. Reviewers are asked to complete a checklist review form and are provided with free text boxes to elaborate on their assessment. These free text comments are reproduced below.

ARTICLE DETAILS

Title (Provisional)

Inequitably Harmed: A Scoping Review Protocol on Patient Safety and Diverse Population Groups

Authors

Ocloo, J; Surendran, Shone; Robin, Blaine; Noel, Leah; Gibson, Jade; Dawson, Shoba

---

VERSION 1 - REVIEW

**Reviewer**	**1**
**Name**	**Mira, José**
**Affiliation**	**Miguel Hernandez University of Elche, Health Psychology**
**Date**	**29-Aug-2025**
**COI**	**None**

The study aims to conduct a scoping review of the literature to identify and synthesize the most relevant evidence on patient safety in disadvantaged or high-risk groups where inequities have been detected or are presumed. The study is relevant and timely, and the methodology described is appropriate. The essential elements of a scoping review are included and explained adequately. Nevertheless, I have several observations that I hope will help the authors to refine their protocol and make it more precise.

First, the examples presented in the opening paragraph of the introduction refer only to the UK. If the scope of the study is broader, as seems to be implied, these references are too limited, and the authors should make an effort to include perspectives from other regions.

Second, although COVID-19 may serve as an illustrative example, I believe its inclusion risks distorting the focus of the study. The pandemic created extraordinary circumstances that are not representative of daily healthcare practice. The issues under study existed before the pandemic and persist afterwards; the justification should not rest primarily on COVID-19 unless the authors can clearly argue otherwise.

Third, it is not clear whether the review will include private healthcare institutions or only public ones. The description appears to be very domestically focused on the UK, while models in Asia or Latin America could differ significantly.

Fourth, the way in which court cases will be explored is not well explained. The meaning of this approach is unclear, and no source is cited that could provide such information—except for the reference to the Ombudsman. Many court cases may not be captured through this route, and clarification is needed.

Fifth, the advisory group, which is presented as a novel element of the approach, is not sufficiently described. The number of members and the recruitment process are not explained. Their role is mentioned only briefly, and it is unclear how their decisions will be applied. For instance, what happens if disagreements arise? The methodology is first described as standardized, and then this additional element is introduced, but the two are not integrated, making the overall method difficult to follow.

Sixth, while scoping reviews generally do not require approval from a research ethics committee, the inclusion of an advisory group complicates this. Involving people as participants—even as peer researchers—may require ethical oversight. This issue should be explicitly addressed, particularly because the document is presented as a protocol.

Seventh, the dissemination plan is limited, and the explanations provided could apply to almost any study or context. A more tailored and specific dissemination strategy would be welcome.

Eighth, the checklist included that results and discussion sections have been reported. Presumably, these will be developed in the future.

Finally, of the 46 references cited, five are authored by the first author. While these may be relevant, the degree of self-citation could be reconsidered to ensure balance.

**Reviewer**	**2**
**Name**	**Martín Delgado, María Cruz**
**Affiliation**	**Hospital Universitario de Torrejón, Hospital Universitario Torrejon**
**Date**	**05-Sep-2025**
**COI**	**None**

---

open-2025-108797

"Inequitably Harmed: A Scoping Review Protocol on Patient Safety and Diverse Population Groups"

We appreciate the opportunity to review this manuscript and commend the authors for addressing such a highly relevant topic. We share the interest in evaluating the impact of

diversity on patient safety, an area where there are still few specific references and where further exploration is much needed. The proposed methodological design appears appropriate to achieve the stated objectives.

However, we believe that several aspects could be clarified or strengthened:

Definition of patient harm: It would be helpful to specify how harm will be assessed in terms of severity and clinical consequences, as this will be essential for conducting the literature review.

Study population: It is not clear whether the review will focus exclusively on adult patients, pediatric patients, or both. We suggest that this be explicitly defined in the protocol.

Updating of references: Many of the cited references are relatively old. We recommend reviewing and, where possible, updating the bibliography with more recent publications that reflect the current state of knowledge in this field.

Overall, this is a timely and relevant proposal that can provide valuable insights once these clarifications are incorporated.

**Reviewer**	**3**
**Name**	**Bellou, Abdelouahab**
**Affiliation**	**Guangdong Academy of Medical Sciences, Emergency Medicine**
**Date**	**21-Sep-2025**
**COI**	**None**

Authors are submitting an interesting scoping review on iniquity and patient safety in the healthcare system.

The subject is of interest and relevant.

Authors must be sure that the scoping review will bring information about the risk of patients safety events related to iniquity of care processes.

Authors must be sure that they used the needed references to demonstrate the innovation of the review.

I attached some of them, authors can analyse.

References:

Exploring patient safety outcomes for people with learning disabilities in acute hospital settings: a scoping review.

Louch G, Albutt A, Harlow-Trigg J, Moore S, Smyth K, Ramsey L, O'Hara JK.BMJ Open. 2021 May 19;11(5):e047102. doi: 10.1136/bmjopen-2020-047102.

The safety of health care for ethnic minority patients: a systematic review. Int J Equity Health. 2020 Jul 8;19(1):118. doi: 10.1186/s12939-020-01223-2.

Ashfaq Chauhan 1, Merrilyn Walton 2, Elizabeth Manias 3, Ramesh Lahiru Walpola 4, Holly Seale 4, Monika Latanik 5, Desiree Leone 5, Stephen Mears 6, Reema Harrison 4

Observed Versus Expected Distribution of Patient

Self-Reported Race and Ethnicity in Quality

Improvement Review Processes at a Single Safety

Net Academic Institution

Delisa Quayson , Meredith Alston , Stefka Fabbri

Racial and Ethnic Disparities in Patient Safety.

Okoroh JS, Uribe EF, Weingart S.J Patient Saf. 2017 Sep;13(3):153-161. doi: 10.1097/PTS.0000000000000133.

Cureus. 2023 Mar 13;15(3):e36090.doi: 10.7759/cureus.36090. eCollection 2023 Mar.

Observed Versus Expected Distribution of Patient Self-Reported Race and Ethnicity in Quality Improvement Review Processes at a Single Safety Net Academic Institution

Diagnosis (Berl). 2025 Mar 3;12(3):358-364.

doi: 10.1515/dx-2024-0160.eCollection 2025 Aug 1.

Equity-Driven Diagnostic Excellence framework: An upstream approach to minimize risk of diagnostic inequity

Noor H Simsam 1, Rawan Abuhamad 2, Khalid Azzam 3

Exploring the connection of health disparities and inequities with health care-acquired infections in North America: A scoping review of the literature.

Tarabay J, Nix CD, Doline K, McClusky J, Catalfumo F, Lewin CA, Gupta R, Robinson K, Bartles R.Am J Infect Control. 2025 Jul;53(7):778-784. doi: 10.1016/j.ajic.2025.04.004. Epub 2025 Apr 10.PMID: 40220799

Bridging diagnostic safety and mental health: a systematic review highlighting inequities in autism spectrum disorder diagnosis.

Srivarathan A, Bradford A, Shearkhani S, Heimlich L, Jefferson S, Miller KE, Smith K, Haskell H, Giardina TD.BMJ Qual Saf. 2025 Aug 25:bmjqs-2025-018723. doi: 10.1136/bmjqs-2025-018723. Online ahead of print.

A Systems-Based Framework for Integrating Health Equity and Patient Safety.

Tsuei J, Bandini JI, Thomas AD, James KF, Etchegaray JM, Schulson L.Jt Comm J Qual Patient Saf. 2025 Jul-Aug;51(7-8):498-506. doi: 10.1016/j.jcjq.2025.04.005. Epub 2025 Apr 22.

Machine learning evaluation of inequities and disparities associated with nurse sensitive indicator safety events.

Georgantes ER, Gunturkun F, McGreevy TJ, Lough ME.J Nurs Scholarsh. 2025 Jan;57(1):59-71. doi: 10.1111/jnu.12983. Epub 2024 May 21.PMID: 38773783

Payer-Based Segregation in Obstetrics and Gynecology Ambulatory Care: Implications for Quality, Safety, and Equity.

Larkin S, Harness E, Arora KS, Qasba N, Chesnokova A, Banks E, George K, Vinekar K.Obstet Gynecol. 2025 May 1;145(5):469-474. doi: 10.1097/AOG.0000000000005883. Epub 2025 Mar 13.

J Am Med Inform Assoc. 2020 Dec 9;27(12):2024-2027.

doi: 10.1093/jamia/ocaa085.

Patient safety and quality improvement: Ethical principles for a regulatory approach to bias in healthcare machine learning

Melissa D McCradden 1, Shalmali Joshi 2, James A Anderson 1 3 4, Mjaye Mazwi 5, Anna Goldenberg 2 6 7 8, Randi Zlotnik Shaul 1 9 10

Inequalities in the Quality and Safety of Post-Diagnostic Primary Care for People With Dementia: A Scoping Review.

Morris C, Techache R, Davies K, Blakeman T, Kontopantelis E, Ashcroft DM, Robinson DL.Int J Geriatr Psychiatry. 2024 Dec;39(12):e70035. doi: 10.1002/gps.70035.

Taking action on inequities: a structural paradigm for quality and safety.

Burra TA, Soong C, Wong BM.BMJ Qual Saf. 2024 May 17;33(6):351-353. doi: 10.1136/bmjqs-2023-017027.

Equity in Pediatric Hospital-Based Safety and Quality Improvement.

Dixon GL, Peña MM, Ellison AM, Johnson TJ.Acad Pediatr. 2024 Sep-Oct;24(7S):S184-S188. doi: 10.1016/j.acap.2024.01.026.PMID: 39428152

Inequities in Inpatient Pediatric Patient Safety Events by Category.

Pantell MS, Karvonen KL, Porter P, Stotts J, Neuhaus J, Bekmezian A.Hosp Pediatr. 2024 Dec 1;14(12):953-962. doi: 10.1542/hpeds.2023-007129.PMID: 39523794

VERSION 1 - AUTHOR RESPONSE

Reviewer comments and responses to them.

Reviewer 1

The study aims to conduct a scoping review of the literature to identify and synthesize the most relevant evidence on patient safety in disadvantaged or high-risk groups where

inequities have been detected or are presumed. The study is relevant and timely, and the methodology described is appropriate. The essential elements of a scoping review are included and explained adequately. Nevertheless, I have several observations that I hope will help the authors to refine their protocol and make it more precise.

#	Section	Issue
1	**Introduction:**	the examples presented in the opening paragraph of the introduction refer only to the UK. If the scope of the study is broader, as seems to be implied, these references are too limited, and the authors should make an effort to include perspectives from other regions.<br><br>**Thank you for this helpful observation, we have now added in several new references as per the tracked changes to reflect the international context.**
2	**Introduction:**	although COVID-19 may serve as an illustrative example, I believe its inclusion risks distorting the focus of the study. The pandemic created extraordinary circumstances that are not representative of daily healthcare practice. The issues under study existed before the pandemic and persist afterwards; the justification should not rest primarily on COVID-19 unless the authors can clearly argue otherwise.<br><br>**Thank you for this comment. We agree and so this text has now been removed.**
3	**Introduction:**	it is not clear whether the review will include private healthcare institutions or only public ones. The description appears to be very domestically focused on the UK, while models in Asia or Latin America could differ significantly.<br><br>**Thank you for this comment and yes, the review will include all papers that meet our inclusion criteria which will include papers regardless of whether they cover private or public institutions.**
4	**Introduction/ Methods**	the way in which court cases will be explored is not well explained. The meaning of this approach is unclear, and no source is cited that could provide such information—except for the reference to the Ombudsman. Many court cases may not be captured through this route, and clarification is needed.<br><br>**Thank you for this comment and we agree that this was not clear. We have therefore clarified the inclusion criteria in the**

		**paper to make clear what legal cases we will be looking at as per below:**<br><br>Landmark legal cases with national/ international patient safety policy impact (e.g., Montgomery v Lanarkshire). General court case judgements will be excluded.
5	**Methods/PPI**	the advisory group, which is presented as a novel element of the approach, is not sufficiently described. The number of members and the recruitment process are not explained. Their role is mentioned only briefly, and it is unclear how their decisions will be applied. For instance, what happens if disagreements arise? The methodology is first described as standardized, and then this additional element is introduced, but the two are not integrated, making the overall method difficult to follow.<br><br>**Thank you for this comment.**<br><br>**We can confirm that the Advisory Group will be consulted on high level issues which have largely been to do with comments on the search strategy. The Advisory Group will not be a part of the core research team working on the review, apart from three peer researchers who have joined the research team from the Advisory Group as researchers. They will be involved in various aspects where they feel they want to be involved or have the time to do this and as part of the research team will be involved in resolving any disagreements on the data.**
6	**Ethics**	while scoping reviews generally do not require approval from a research ethics committee, the inclusion of an advisory group complicates this. Involving people as participants—even as peer researchers—may require ethical oversight. This issue should be explicitly addressed, particularly because the document is presented as a protocol.<br><br>**Thank you for this comment. We can confirm that our peer researchers are involved in this review as patient and public involvement (PPI) members and not as participants. Involving PPI members in reviews is now common and we do not believe this requires ethics approval.**

Formatted: Font: (Default) Aptos

Formatted: Font: (Default) Aptos

7	**Dissemination**	the dissemination plan is limited, and the explanations provided could apply to almost any study or context. A more tailored and specific dissemination strategy would be welcome.<br><br>**Thank you. We have now added some extra information to be clearer about our intentions as per below:**<br><br>**The final scoping review paper will be disseminated through publication in a peer-reviewed journal and will be presented to various stakeholders. This will include to Integrated Care Boards (ICBs) who are NHS organizations responsible for planning and delivering local health services within a specific geographic area and to the National Institute for Health Research Applied Research Collaborations (who support local partnerships for applied health and care research) connected to the work and their considerable community and voluntary networks.**<br><br>**The PI for the study has various patient safety network contacts which will allow the protocol to be disseminated: via NHS England (the organization that oversees the provision of healthcare services in England, which is part of the United Kingdom's overall National Health Service (NHS), harmed patients and their families and organisations supporting patients in this area and via the WHO Global Patient Safety Network which the PI is a member of.**
8	**Discussion**	the checklist included that results and discussion sections have been reported. Presumably, these will be developed in the future.<br><br>**Thank you for your comment. The checklist has been amended to reflect that what we are reporting in the results and discussion section is to do with the protocol. The methodology described in the results section is a reflection of how the results will be described in the full review paper.**
9	**References**	of the 46 references cited, five are authored by the first author. While these may be relevant, the degree of self-citation could be reconsidered to ensure balance.<br><br>**Thank you. Two of these citations have been removed. We have left in the remaining three as we feel they are important unique contributions in the context of this discussion.**

Formatted: Font: 11 pt, Bold, Complex Script Font: Arial, 11 pt, Bold

Formatted: Font: 11 pt, Complex Script Font: Arial, 11

Formatted: Font: (Default) Aptos, 11 pt, Bold, Complex Script Font: 11 pt, Bold

Formatted: Font: (Default) Aptos, 11 pt, Bold, Complex Script Font: 11 pt, Bold

Formatted: Font: (Default) Aptos, 11 pt, Bold, Complex Script Font: 11 pt, Bold

Formatted: Font: (Default) Aptos, 11 pt, Bold, Complex Script Font: 11 pt, Bold

Formatted: Font: (Default) Aptos, 11 pt, Bold, Complex Script Font: 11 pt, Bold

Formatted: Font: (Default) Aptos, 11 pt, Bold, Complex Script Font: 11 pt, Bold

Formatted: Font: (Default) Aptos, 11 pt, Bold, Complex Script Font: 11 pt, Bold

Formatted: Font: (Default) Aptos, 11 pt, Bold, Complex Script Font: 11 pt, Bold

Formatted: Font: (Default) Aptos, 11 pt, Bold, Complex Script Font: 11 pt, Bold

Formatted: Font: (Default) Aptos, 11 pt, Bold, Complex Script Font: 11 pt, Bold

Formatted: Font: (Default) Aptos, 11 pt, Bold, Complex Script Font: 11 pt, Bold

Formatted: Font: (Default) Aptos, 11 pt, Bold, Complex Script Font: 11 pt, Bold

Formatted: Font: (Default) Aptos, 11 pt, Bold, Complex Script Font: 11 pt, Bold

Formatted: Font: (Default) Aptos, 11 pt, Bold, Complex Script Font: 11 pt, Bold

Reviewer 2

#	Section	Issue
1	**Introduction/ Background**	Definition of patient harm: It would be helpful to specify how harm will be assessed in terms of severity and clinical consequences, as this will be essential for conducting the literature review.<br><br>**Thank you for this comment. Our research questions seek to identify what evidence exists on patient safety harm (mental and physical). In the process of data extraction, we will extract key results and evidence on what has been identified as harm and how these impact on different population groups and in which clinical area. To do this we have added the wording to our DE form: It would be helpful to record key results in terms of severity in the form of disparities that exists for different groups and record the clinical consequences relating to those disparities.**<br><br>**We believe this will be the most feasible way of addressing the comments that you have made.**
2	**Methods**	Study population: It is not clear whether the review will focus exclusively on adult patients, pediatric patients, or both. We suggest that this be explicitly defined in the protocol.<br><br>**Thank you. I can confirm that we will be looking at all population demographics adult and paediatrics**
3	**References**	Updating of references: Many of the cited references are relatively old. We recommend reviewing and, where possible, updating the bibliography with more recent publications that reflect the current state of knowledge in this field.<br><br>**Thank you. We have now reviewed the references and have included several new ones related to the international literature, recent reports on patient safety and patient harm and have included many of the helpful references suggested by the reviewers in relation to equity and harm.**

Reviewer 3

#	Section	Issue
1	**Methods**	Authors must be sure that the scoping review will bring information about the risk of patients safety events related to iniquity of care processes.<br><br>**Thank you for your comment. We are confident that our research questions and inclusion criteria will be sufficient to**

		**identify the papers/information needed on inequities in relation to patient safety events.**
2	**References**	Authors must be sure that they used the needed references to demonstrate the innovation of the review.<br><br>**Thank you. We believe we have addressed the issue of references in this protocol and the scoping review itself once conducted will highlight many other relevant papers for citation in the main paper.**

Exploring patient safety outcomes for people with learning disabilities in acute hospital settings: a scoping review.
Louch G, Albutt A, Harlow-Trigg J, Moore S, Smyth K, Ramsey L, O'Hara JK.BMJ Open. 2021 May 19;11(5):e047102. doi: 10.1136/bmjopen-2020-047102.

The safety of health care for ethnic minority patients: a systematic review. Int J Equity Health. 2020 Jul 8;19(1):118. doi: 10.1186/s12939-020-01223-2.
Ashfaq Chauhan 1, Merrilyn Walton 2, Elizabeth Manias 3, Ramesh Lahiru Walpola 4, Holly Seale 4, Monika Latanik 5, Desiree Leone 5, Stephen Mears 6, Reema Harrison 4

Observed Versus Expected Distribution of Patient
Self-Reported Race and Ethnicity in Quality
Improvement Review Processes at a Single Safety
Net Academic Institution
Delisa Quayson , Meredith Alston , Stefka Fabbri
Racial and Ethnic Disparities in Patient Safety.
Okoroh JS, Uribe EF, Weingart S.J Patient Saf. 2017 Sep;13(3):153-161. doi: 10.1097/PTS.0000000000000133.

Cureus. 2023 Mar 13;15(3):e36090.doi: 10.7759/cureus.36090. eCollection 2023 Mar.
Observed Versus Expected Distribution of Patient Self-Reported Race and Ethnicity in Quality Improvement Review Processes at a Single Safety Net Academic Institution
Diagnosis (Berl). 2025 Mar 3;12(3):358-364.
doi: 10.1515/dx-2024-0160.eCollection 2025 Aug 1.

Equity-Driven Diagnostic Excellence framework: An upstream approach to minimize risk of diagnostic inequity
Noor H Simsam 1, Rawan Abuhamad 2, Khalid Azzam 3

Exploring the connection of health disparities and inequities with health care-acquired infections in North America: A scoping review of the literature.
Tarabay J, Nix CD, Doline K, McClusky J, Catalfumo F, Lewin CA, Gupta R, Robinson K, Bartles R.Am J Infect Control. 2025 Jul;53(7):778-784. doi: 10.1016/j.ajic.2025.04.004. Epub 2025 Apr 10.PMID: 40220799

Bridging diagnostic safety and mental health: a systematic review highlighting inequities in autism spectrum disorder diagnosis.
Srivarathan A, Bradford A, Shearkhani S, Heimlich L, Jefferson S, Miller KE, Smith K, Haskell H, Giardina TD.BMJ Qual Saf. 2025 Aug 25:bmjqs-2025-018723. doi: 10.1136/bmjqs-2025-018723. Online ahead of print.

A Systems-Based Framework for Integrating Health Equity and Patient Safety.
Tsuei J, Bandini JI, Thomas AD, James KF, Etchegaray JM, Schulson L.Jt Comm J Qual Patient Saf. 2025 Jul-Aug;51(7-8):498-506. doi: 10.1016/j.jcjq.2025.04.005. Epub 2025 Apr 22.

Machine learning evaluation of inequities and disparities associated with nurse sensitive indicator safety events.
Georgantes ER, Gunturkun F, McGreevy TJ, Lough ME.J Nurs Scholarsh. 2025 Jan;57(1):59-71. doi: 10.1111/jnu.12983. Epub 2024 May 21.PMID: 38773783

Payer-Based Segregation in Obstetrics and Gynecology Ambulatory Care: Implications for Quality, Safety, and Equity.
Larkin S, Harness E, Arora KS, Qasba N, Chesnokova A, Banks E, George K, Vinekar K.Obstet Gynecol. 2025 May 1;145(5):469-474. doi: 10.1097/AOG.0000000000005883. Epub 2025 Mar 13.

J Am Med Inform Assoc. 2020 Dec 9;27(12):2024-2027.
doi: 10.1093/jamia/ocaa085.
Patient safety and quality improvement: Ethical principles for a regulatory approach to bias in healthcare machine learning
Melissa D McCradden 1, Shalmali Joshi 2, James A Anderson 1 3 4, Mjaye Mazwi 5, Anna Goldenberg 2 6 7 8, Randi Zlotnik Shaul 1 9 10

Inequalities in the Quality and Safety of Post-Diagnostic Primary Care for People With Dementia: A Scoping Review.
Morris C, Techache R, Davies K, Blakeman T, Kontopantelis E, Ashcroft DM, Robinson DL.Int J Geriatr Psychiatry. 2024 Dec;39(12):e70035. doi: 10.1002/gps.70035.

Taking action on inequities: a structural paradigm for quality and safety.

Burra TA, Soong C, Wong BM.BMJ Qual Saf. 2024 May 17;33(6):351-353. doi: 10.1136/bmjqs-2023-017027.

Equity in Pediatric Hospital-Based Safety and Quality Improvement.
Dixon GL, Peña MM, Ellison AM, Johnson TJ.Acad Pediatr. 2024 Sep-Oct;24(7S):S184-S188. doi: 10.1016/j.acap.2024.01.026.PMID: 39428152

Inequities in Inpatient Pediatric Patient Safety Events by Category.
Pantell MS, Karvonen KL, Porter P, Stotts J, Neuhaus J, Bekmezian A.Hosp Pediatr. 2024 Dec 1;14(12):953-962. doi: 10.1542/hpeds.2023-007129.PMID: 39523794

VERSION 2 - REVIEW

**Reviewer**	**1**
**Name**	**Mira, José**
**Affiliation**	**Miguel Hernandez University of Elche, Health Psychology**
**Date**	**31-Oct-2025**
**COI**	

Authors have already improved the manuscript. Comments and insights have been considered and responses are clear

**Reviewer**	**2**
**Name**	**Martín Delgado, María Cruz**
**Affiliation**	**Hospital Universitario de Torrejón, Hospital Universitario Torrejon**
**Date**	**15-Nov-2025**
**COI**	

We thank the authors for the changes made to the manuscript, taking into account the reviewers' suggestions.
